# Collaborative AI for precision neurorehabilitation: a roadmap

**DOI:** 10.1186/s12984-025-01810-w

**Published:** 2025-11-27

**Authors:** Sook-Lei Liew, R. James Cotton, Etienne Burdet, Sergi Bermúdez i Badia, Pablo Celnik, James H. Cole, Ruishan Liu, Surjo R. Soekadar, Carolee Winstein, Nicolas Schweighofer

**Affiliations:** 1 Mark and Mary Stevens Neuroimaging and Informatics Institute, Keck School of Medicine, Department of Neurology, Chan Division of Occupational Science and Occupational Therapy, Department of Biomedical Engineering, University of Southern California, 2025 Zonal Ave, Los Angeles, CA 90033 USA; 2 Department of Physical Medicine and Rehabilitation, Northwestern University, 710 N. Lake Shore Drive, Chicago, IL 60611 USA; 3 Department of Bioengineering, Imperial College of Science, Technology and Medicine, White City campus, London, SW7 2AZ UK; 4 Faculdade de Ciências Exatas e da Engenharia & NOVA LINCS, Universidade da Madeira Campus da Penteada, 9020-105 Funchal, Portugal; 5 Agência Regional para o Desenvolvimento de Investigação, Tecnologia e Inovação (ARDITI), Campus da Penteada, 9020-105 Funchal, Madeira Portugal; 6 Shirley Ryan AbilityLab, 355 East Erie ST, Chicago, Il 60611 USA; 7 Hawkes Institute, Department of Computer Science, University College London, 90 High Holborn, London, WC1V 6LJ UK; 8 Department of Computer Science, University of Southern California, 1031 Downey Way, Los Angeles, CA 90089 USA; 9 Clinical Neurotechnology Lab, Dept. of Psychiatry and Neurosciences, Charité Campus Mitte (CCM), Charité - Universitätsmedizin Berlin, Charitéplatz 1, 10117 Berlin, Germany; 10 Biokinesiology and Physical Therapy, University of Southern California, 1540 Alcazar St, Los Angeles, CA 90033 USA

**Keywords:** Artificial intelligence, Collaborative AI, Neurorehabilitation, Precision medicine, Bayesian modeling, Digital twin, Large language models, Reinforcement learning

## Abstract

Precision rehabilitation seeks to improve care for individuals by identifying personalized treatments that enhance both the efficacy and efficiency of care. Rapid advances in artificial intelligence (AI) and data-driven methodologies stand to greatly enhance precision rehabilitation methods, particularly when paired with clinical expertise and patient-driven goals. Although AI has been successfully integrated into healthcare in fields such as precision oncology, the dynamic, multi-faceted, complex nature of rehabilitation require a different approach than standard predictive models. Here, we argue that precision rehabilitation may benefit from *collaborative AI*, in which humans and AI work together with AI distilling complex data that humans can use as part of their nuanced decision-making process. We review the current landscape of precision rehabilitation and explore how collaborative AI, and AI in general, can advance the field. We begin by outlining a roadmap for a general collaborative AI system in rehabilitation, noting four key challenges that must be addressed. Next, we examine three existing precision rehabilitation frameworks that have been developed independently by several of the co-authors, which share four common key elements of the roadmap. We then describe examples of how AI has already been applied to specific aspects of complex rehabilitation scenarios, as these may be integrated into larger collaborative AI models. We follow this with a discussion of how to make AI-based precision rehabilitation a reality, with an emphasis on the large data required for these models to make accurate predictions, as well as potential ethical issues. Finally, we conclude with recommendations for future directions. Ultimately, collaborative AI promises to transform rehabilitation by leveraging vast, diverse datasets to create individualized digital profiles, which can first be used to simulate the effects of rehabilitation interventions in silico, maximizing impacts before real-world implementation. By merging personalized rehabilitation strategies based on empirical evidence with the depth and complexity of clinical knowledge and reasoning, collaborative AI holds promise as a powerful tool to help clinicians advance patient care and improve long-term outcomes.

## Introduction: the current challenges of rehabilitation

The overarching goal of medical rehabilitation is to improve health, well-being, and quality of life by preventing or minimizing the impact of disabilities. Determining the optimal intervention strategy based on an individual’s clinical profile has been recognized as one of the most pressing challenges in rehabilitation [1–5]. *Precision rehabilitation* seeks to address this challenge by personalizing treatment to improve efficacy and efficiency of rehabilitation for each individual [3]; personalized methods stand to benefit from the integration of artificial intelligence (AI) and data-driven methodologies with clinical expertise and patient-driven goals [5, 6]. While we focus specifically on neurorehabilitation in this paper, the underlying principles extend broadly across various areas of medical rehabilitation research.

Delivering effective precision rehabilitation is difficult because of four major complexities: (1) diverse patient-centered outcomes, (2) high between-patient variability, (3) many possible treatments, each with varying possible doses, timelines, and outcomes, and (4) the large, high dimensional and multimodal data needed to predict patient outcomes. We now briefly review these complexities.

First, the ultimate goal of the rehabilitation process is determined by the clinical care team, in dialogue with the patient, who together select meaningful outcomes. This approach is crucial because it ensures the treatment plan aligns with the patient’s objectives, values, and real-world constraints. However, the collaborative selection of meaningful, nuanced outcome(s) leads to wide-ranging variability in the recovery target, such as regaining arm function, reducing fall risk during walking, or increasing participation in meaningful activities. Compared to predicting a *hazard ratio* for survival, such as is done in precision medicine for cancer, the goal of precision rehabilitation is to identify a *functional outcomes ratio* that optimizes treatment to preserve overall quality of life. This introduces challenges to predictive modeling because rehabilitation plans may need to be optimized to predict multiple goals, many of which are difficult to quantify.

Second, rehabilitation is characterized by large between-patient variability, making it difficult to predict how patients will respond to different treatments. Personal factors, such as the patient’s current and premorbid physical abilities, the type and location of any nervous system injury, time since injury, age, self-motivation, mood, cognition, fatigue, sleep, social support, and social determinants of health (e.g., socioeconomic status, education, access to transportation), along with the patient’s real-world engagement outside of therapy and actual activity during therapy (amount, duration, repetitions), all significantly influence rehabilitation outcomes. These many heterogeneous factors make it challenging to predict how a given patient will respond to a specific treatment plan. Indeed, the number of combinations of variables that need to be considered to predict outcomes can be astonishingly large. For instance, there are over 1 million possible linear predictive models if just 20 predictors are considered without interactions (2^20^). While such a prediction task is extremely hard for the human brain, it is a relatively simple task for an AI or machine learning (ML) system—assuming the existence of large, high-quality training data (see the fourth point, below).

Third, rehabilitation interventions are often delivered sequentially over periods of months, with delayed improvements in outcomes. Interventions are also often multi-faceted with many potential active ingredients (e.g., motor skill training, transcranial magnetic stimulation, drugs, clinician interactions, or even surgery) that can be modulated in dose and time and are tracked with diverse outcome measures. As a result, there are many potential treatment plans, given the multiple scheduling factors that modulate recovery, the high between-patient variability, and the multiple scheduling patient constraints and preferences. For instance, even the seemingly simple task of deciding whether to apply or not the same unimodal treatment each week for 6 months (26 weeks) results in ~ 67 million possible treatment plans. AI, in collaboration with the clinician, can play a valuable role in identifying and recommending treatment plans that have the best chance of maximizing recovery, while prioritizing the patient’s preferences and constraints.

Fourth and finally, to accurately predict patient outcomes, we need large, multimodal, and diverse datasets that capture the wide variability across patients, treatment goals, treatments, and outcomes. Although such datasets do not currently exist, efforts to harmonize data within specific conditions and to provide data standards for newly collected data are beginning to emerge and are discussed in detail below (See "Making AI-Based Precision Rehabilitation a Reality").

These four complexities distinguish the field of rehabilitation from many other areas of medicine, where outcomes are often narrowly defined (e.g., liver function tests for hepatic insufficiency) and influenced by a limited set of quantifiable factors (e.g., whether or not to deliver a pharmacologic intervention). One of the best examples of precision medicine is in oncology, where high-throughput technologies, such as DNA and RNA sequencing, have been leveraged to profile tumors, predict therapeutic responses, and inform cancer treatment decisions [7]. Large-scale data sharing efforts like The Cancer Genome Atlas [8] and the Flatiron Health Database [9] integrate genomic, clinical, and outcome data across multiple cancers. These datasets are used to predict mutation-drug interactions, stratify patients, identify optimal therapies, and evaluate off-label drug use to extend treatment options beyond FDA guidelines [10–12]. AI-based computational tools can also be used to analyze these large, multimodal datasets to refine participant eligibility for clinical trials, ensuring broader patient inclusion without compromising safety or efficacy. The success of these initiatives illustrates the need for, and the benefits of, high-quality, integrated large datasets for advancing personalized treatment, particularly when the factors and outcomes at play are well characterized.

In contrast, rehabilitation’s dynamic, multi-faceted nature may require a different approach than the standard predictive models applied to other, more straightforward areas of healthcare. We argue that precision rehabilitation may specifically benefit from *collaborative AI, *which refers to humans and AI working together to achieve what neither can accomplish alone, with AI distilling complex data that humans can then use as part of their nuanced decision-making process [13–15]. In this paper, we will review the current landscape of precision rehabilitation and explore how collaborative AI, and AI in general, can advance this field. First, we will outline a roadmap for a general collaborative AI system in rehabilitation. Next, we examine three existing precision rehabilitation frameworks that have been developed independently by several of the co-authors, which share common key elements of the roadmap [5, 6, 16]. Then, we describe examples of how AI has already been applied to complex rehabilitation scenarios. We follow with a discussion of how to make AI-based precision rehabilitation a reality, with an emphasis on the large data required along with ethical issues. Finally, we conclude with recommendations for future directions.

## A roadmap for collaborative AI in rehabilitation

### Collaborative AI defined

Human-AI collaboration, or *collaborative AI*, is an evolving concept initially introduced by J.C.R. Licklider in 1960 through the notion of “Man-Computer Symbiosis” [17]. Licklider envisioned a future where humans and computers would operate together in a mutually beneficial partnership, such that the strengths of both are leveraged to achieve superior outcomes. Rather than replacing humans in specific tasks, AI acts as a complementary force, augmenting human expertise to improve processes and decision-making through iterative, interactive collaboration. This type of partnership typically involves humans contributing creativity, critical thinking, safety, responsibility, and context, while the AI excels at learning from data, automating tasks, and identifying patterns in high-dimensional inputs. A well-known example of this collaboration emerged in 1998, a year after IBM’s Deep Blue defeated world chess champion Garry Kasparov. This landmark win led to a period of innovative collaboration in which teams of humans and computers working together, termed Centaurs, became the world’s top chess players [18, 19]. Here, we posit that collaborative AI can enhance the rehabilitation ecosystem by providing clinicians with data-driven insights to inform more effective and personalized rehabilitation plans.

### The four key components of a collaborative AI rehabilitation system

**Fig. 1:**
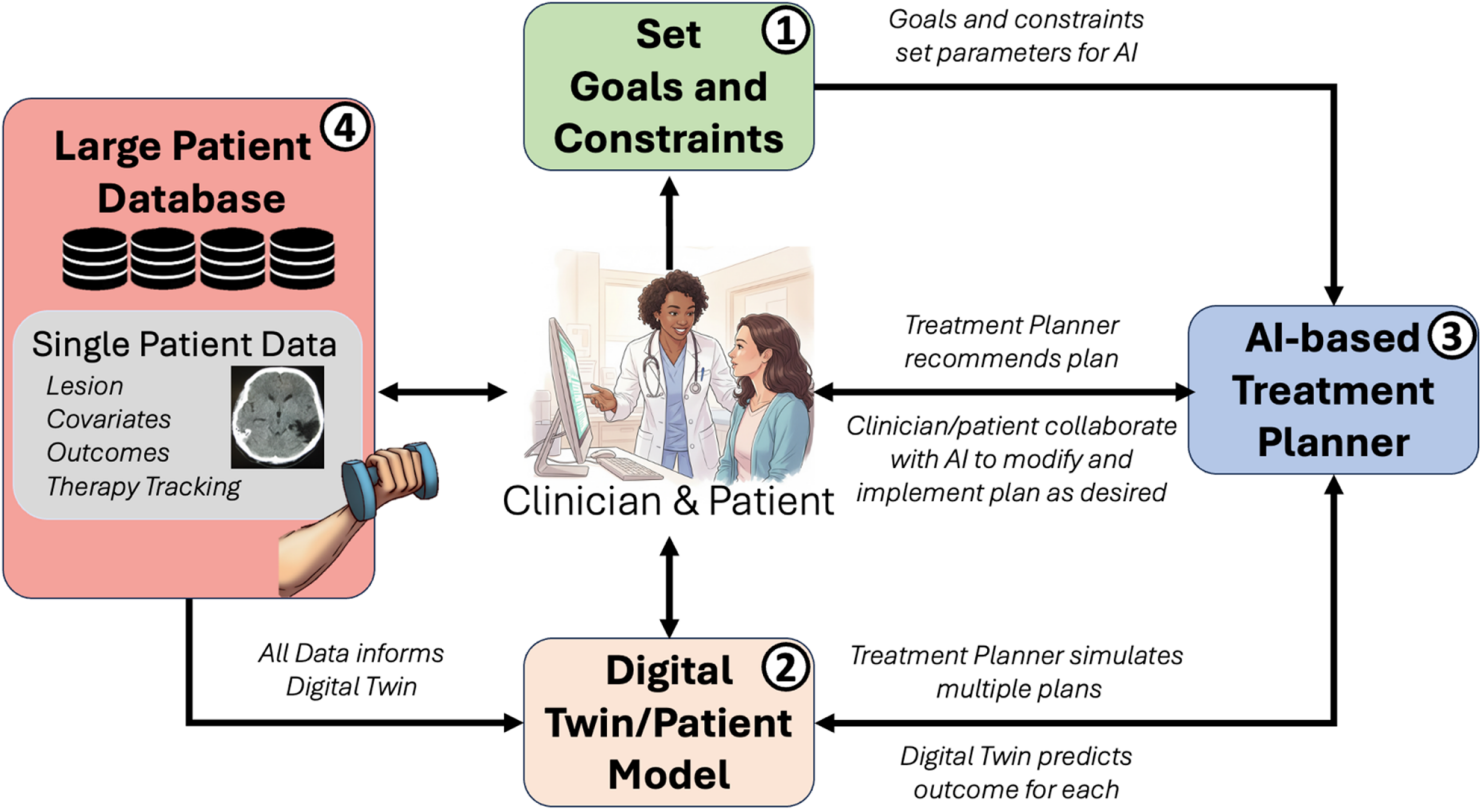
A proposed collaborative AI rehabilitation system with four main components; see text for details. Illustrative components generated using Imagen (Google, Gemini v2.5)

Precision rehabilitation can be viewed as a sequential decision-making problem in which the clinician-AI team interacts with the patient iteratively to identify an optimal treatment plan, out of a large space of possible interventions, in order to maximize long-term functional outcomes. A precision neurorehabilitation system capable of generating an optimal treatment plan has four main components, as alluded to in the introduction, and as we proposed previously [5]—see Fig. 1:

(1) A collaborative AI goal-setting system to define the goals, constraints, and preferences based on input from both the patient and clinician;

(2) A patient-specific predictive model, often called the “patient twin” or “digital twin”, which predicts outcomes for an individual, given interventions based on personalized model parameters;

(3) An AI-based intelligent decision-making system that recommends treatment plans to maximize patient-defined goals using the predictive model; and

(4) A large, multi-modal dataset from the current patient and thousands of other patients that informs all aspects of the model.

#### Component 1: collaborative goal-setting between the AI system, the clinician, and the patient

The collaborative goal-setting system component will set parameters for the AI algorithm based on the clinician’s judgment and the patient’s preferences. We envision two primary ways in which the clinician and the AI system can collaborate to iteratively set and adapt the goals of rehabilitation by: (1) defining the treatment goals (operationalized in our model as the reward function) and (2) setting constraints and preferences.

First, the primary treatment goals are aligned into a *reward function* that guides the AI system to learn the optimal behavior that leads to the desired outcome. Collaborative AI systems have their roots in two related engineering fields—optimal control and reinforcement learning—which are used to determine sequences of actions that maximize outcomes. In both cases, the cost (optimal control) and reward (reinforcement learning) functions are crucial components that assign a numerical value (henceforth, the reward) to each action taken by the agent in a particular state. The reward indicates how good or bad that action was in terms of achieving the desired goal. In rehabilitation, the primary treatment goal (reward function) is defined by the clinician and patient based on the patient’s objectives and meaningful outcomes. For instance, in our recent preliminary work, the reward function was a function of an upper extremity outcome score, with equal weighting for short- and long-term outcomes [5]. If the emphasis is on long-term recovery of arm and hand function, the reward function can be further adjusted to put equal emphasis on distal versus proximal upper extremity functions. In any case, the treatment plan will depend on the choice of the outcome measures and the terms and weights of the reward function, which can be adjusted. After any adjustments, the algorithm will be rerun, and the clinician will be able to visualize the proposed plan and forecasted outcomes (see component 2 below) and modify the plan as desired.

Second, not all treatment plans are feasible or desirable. The clinician and patient can also place constraints upon the AI-generated treatment plans, such as maximum feasible total doses, patient schedule changes due to unexpected events (e.g., an infection or fatigue), as well as limitations due to reimbursement systems and access to therapy. Other common clinical constraints include high activity-dependent fatigability [20], comorbid conditions, patient schedule changes (e.g., travel), socioeconomic and interpersonal needs, and reimbursement needs. Finally, patient preferences in scheduling and task practice are important to maximize motivation and gains in rehabilitation [21, 22]. Thus, the treatment plan must consider all scheduling constraints, which we have proposed to classify into clinical constraints, logistical constraints, and patient preferences [5]. Through collaboration with the AI system, the clinician and patient can input “hard” constraints, such as the minimum or maximum daily practice doses every two weeks, and, if needed, “soft” constraints, by modifying the weights of the reward function.

#### Component 2: a patient predictive “twin” model that can be simulated to predict outcomes in different scenarios

Patient “digital twin” modeling is a powerful approach to personalize healthcare and optimize treatment strategies [5, 6]. A patient digital twin is a dynamic, virtual representation of a patient that integrates a wide range of health-related data, including biological factors such as impairment level, lesion size and location, medical history and demographics, and even real-time sensor data. This detailed model enables both clinicians and AI-based decision-makers to simulate the impact of different treatment options on the desired outcomes for an individual patient at any given time. Crucially, this model will be trained both from the incoming data of the current patient and from data in a large rehabilitation database, which will contain clinical records, including treatments and responses, and clinically relevant covariates of thousands of other patients [5] (see Component 4 below). Upon patient intake, baseline clinical and demographic variables will be inputted to generate initial long-term predictions. However, because of the large between-patient variability in response to treatment, these initial predictions will not be highly accurate. Thus, the model will be updated iteratively during the course of treatment, with the clinician updating the dataset with the patient’s own data (see below), allowing more accurate and precise long-term predictions. Thus, the digital twin model is a crucial component of a collaborative AI system as it forms the basis for testing all plans generated by the AI decision-making algorithm (see below). It also allows the clinician and patient to visualize the long-term predicted outcomes in response to multiple different scenarios. For instance, a clinician could test the effect of modifying the reward function, changing constraints and preferences (e.g., different values of a covariate representing fatigue levels during therapy), or compare different rehabilitation schedules [5].

#### Component 3: an AI-based intelligent decision-making algorithm that recommends optimal treatment plans

A collaborative AI system must help in the planning process through the exponentially large space of treatment decisions by maximizing long-term rewards (goal achievement) while accounting for new information as it becomes available. This is formalized as an AI agent that iteratively generates optimal treatments through interactions with the digital patient: the agent tries different treatments, the digital twin generates different predicted outcomes, which are graded by the reward model, the agent adjusts its strategy accordingly so that better treatments are more likely to be selected, and real-life information is fed back to the model to further improve the model as the patient moves through their actual rehabilitation process.

Prior research has identified several AI/ML-based systems that could provide personalized treatment plans: (1) Reinforcement Learning (RL), a branch of machine learning, offers a way to enhance decision-making by learning optimal strategies through trial and error and addressing sequential decision-making challenges. (2) Unsupervised learning could be used to find clusters of patients with similar factors and identify the treatment plans that led to the best outcomes, while (3) semi-supervised learning uses both labeled and unlabeled data to train models, leveraging vast amounts of available data, even if only a fraction is labeled. The optimal goal of these learning methods is to determine the Optimal Dynamic Treatment Regimes (ODTRs) [23], which is the learned optimal decision rule (which arises from the AI agent and the digital twin).

#### Component 4: a large multi-modal database of rehabilitation-relevant data processed by AI to inform both the digital patient model and intelligent decision-making system

To infer the optimal intervention, the collaborative AI system will need to ensure that patient models are built upon large, heterogeneous, and generalizable databases of previous patient data that can accurately extrapolate to the current patient and be refined with incoming data from this patient. Large, diverse, reliable data sources are thus required to inform the AI systems, including the creation of the digital twin and the decision-making algorithm. As the database expands with each new patient, the model can evolve to incorporate additional factors that will further support informed decision-making. In addition, iterative monitoring, evaluating, and reassessing will be critical to improve long-term predictions for the current patient with updated outcomes, treatment variables, clinical variables, and even goals during treatment. The clinician should augment any digital data by continuously assessing factors such as mood, cognition, self-motivation, social support, fatigue, sleep, and social determinants of health (SDoH), which are hard to model and can fluctuate. Such integration of existing data with real-time incoming data is critical for the success of this approach. Although such large data sources and data infrastructures do not currently exist, emerging efforts to generate these are growing (see "Making AI-based precision rehabilitation a reality").

## AI-based precision rehabilitation frameworks

As outlined above, significant challenges must be addressed to develop and implement intelligent, personalized, collaborative AI systems. In particular, there is a need to identify, adapt, and develop new and appropriate techniques for intelligent decision-making to support the complex and unique combination of variables germane to precision rehabilitation. However, recent research has introduced several promising precision rehabilitation frameworks aimed at predicting outcomes and optimizing therapy. Notably, some of us have proposed frameworks that, while developed independently, share the four common elements discussed above (Fig. 1). Below, we briefly review the three proposed frameworks: (1) The Causal Framework for Precision Rehabilitation lays the foundation for a framework that integrates multimodal data into a causal model of patient recovery that can then be used to optimize rehabilitation in multiple domains [6]. (2) The NeuroAIreh@b framework uses machine learning to assess the patient’s functional status and select appropriate therapy tasks [16]. This system is currently being tested in a clinical trial, and has been refined with patient data acquired in a preliminary feasibility study [24]. (3) Finally, precision rehabilitation via the Contextual Model-Based Reinforcement Learning framework shows how the patient model can be used within model-based reinforcement learning to optimize rehabilitation treatment schedules given the between-patient variability and constraints in possible treatments [5].

### A causal framework for precision rehabilitation (CFPR)

The CFPR seeks to model diverse real-world rehabilitation data in order to improve long-term functional outcomes for individual patients by providing data-driven recommendations of the optimal interventions based on detailed multimodal measurements from those patients [6]. This framework leverages the growing field of causal inference [25, 26] to build causal models that describe the process of rehabilitation. The causal models are formal articulations of hypotheses about how different factors influence each other and ultimately patient function. In this sense, the CFPR builds on Computational Neurorehabilitation, which emphasizes computational modeling of neuroplasticity during rehabilitation [27]. The CFPR advances this approach by linking detailed impairment measurements to functional activities of daily living and participation [28].

Causal inference provides the mathematical framework for fitting these models to large, heterogeneous datasets that include detailed data on interventions, as well as the tools to make counterfactual inferences about how a patient would have responded to alternative interventions. The CFPR focuses on building the causal models using structured causal diagrams, where components of the overall model are specific causal mechanisms that can be tested independently or by combining heterogeneous datasets from different sites [29].

A key challenge in precision neurorehabilitation is predicting neuroplastic changes resulting from different interventions. This requires causal models that can represent intervention effects while being constrained by available data. However, interventions are rarely documented with sufficient detail. One promising approach to documenting interventions is the Rehabilitation Treatment Specification System (RTSS) [30, 31], which structures interventions into active ingredients, target effects, and hypothesized mechanisms of action. Causal models of neuroplasticity and rehabilitation can then be fit to longitudinal data of rehabilitation outcomes at different levels of granularity (kinematics and kinetics, imaging, omics, impairment, participation, social determinants of health, and interventions documented via the RTSS). These causal models, trained on longitudinal rehabilitation outcomes across multiple data levels (e.g., imaging, impairment, participation, social determinants), can serve as patients’ digital twins to optimize interventions and maximize long-term functional recovery.

The CFPR also outlines how these digital twins can be used to simulate the outcomes after counterfactual interventions for different patients at different points along their recovery trajectory to identify a decision support tool that suggests the optimal intervention at each point in time. This intervention policy is an example of learning the ODTR [23]. The CFPR can be mapped onto the four proposed components (Fig. 1) with the causal models being the predictive digital twins (component 2), which is learned from large datasets (component 4), and the ODTR being the AI decision agent (component 3), which can be optimized for patient-selected goals (component 1).

### The NeuroAIreh@b framework

The NeuroAIreh@b framework addresses limitations of conventional neurorehabilitation tools, which often struggle to adapt to the unique needs of each patient and require extensive manual assessment [16]. This AI-driven framework personalizes rehabilitation activities by relying on patient profiling, task selection, and progress monitoring across cognitive, motor, emotional, and social domains. It consists of three main modules:

1. *Adaptive Digital Patient Modeling*: a Predicted Profile is created via four components: (i) *Assessment Instruments*, where clinicians use validated tools to evaluate each of the cognitive, motor, emotional, and social domains. ML is used to structure this baseline data for monitoring longitudinal progress into a formal language, facilitating the analysis of patient profiles. (ii) *Assessed Functional Profiles (AFP)*, where data from various assessment tools and socio-demographic inputs are aggregated into a cohesive AFP using ML. (iii) *Normative Comparisons*: the AFP is contextualized against normative demographic and clinical data to establish a reference baseline, enabling an objective measure of a patient’s functional status. (iv) *Predicted Profile Update*: As model prediction errors and tracking drifts occur, calibration uses ML to refine the patients’ profile and adjust for any discrepancies.
2. *Training Selection*: once the patient’s baseline profile, or digital twin, is established, NeuroAIreh@b selects customized training tasks based on specific rehabilitation needs. (i) *Setting Training Objectives*: AFP rehabilitation objectives are translated into quantifiable parameters, enabling clinicians to create measurable, structured goals for each domain. (ii) *Task Selection*: Suitable training tasks that align with the AFP and target specific domain requirements are selected, with difficulty adjusted as necessary. (iii) *Initial Task Parameters*: Based on the AFP, ML algorithms establish initial task settings by analyzing the relationships between task profiles and patient profile parameters, ensuring that tasks are challenging yet achievable.
3. *Training Session Execution*: during each training session, NeuroAIreh@b delivers virtual reality-based training tasks to continuously adapt task difficulty based on real-time patient performance, maintaining engagement and ensuring effective training. NeuroAIreh@b monitors performance to keep the patient’s success rate between 50% and 70%, thus avoiding boredom or frustration; an AI algorithm dynamically adjusts task difficulty in real-time, maintaining an optimal challenge level based on the patient’s ongoing performance.

The NeuroAIreh@b framework aligns well with the four components of a collaborative AI precision rehabilitation system, leveraging adaptive patient modeling, real-time predictive updates, intelligent treatment planning, and large-scale data integration: (1) Collaborative Goal Setting System is achieved by aggregating clinician assessments into an Assessed Functional Profile (AFP) and refining goals through AI-driven calibration. (2) Patient Predictive Model (“Digital Twin”) is accomplished via the continuously updated Predicted Profile to track progress, mirroring the patient twin concept for real-time adaptation. (3) Intelligent Decision-Making Treatment Planning is performed by the algorithm that dynamically selects and adjusts training tasks, optimizing challenge levels based on AFP and performance data. (4) Multi-Modal Data Integration is represented throughout NeuroAIreh@b, which contextualizes patient data against normative datasets.

### Precision rehabilitation via contextual model-based reinforcement learning

Finally, in recent work, we proposed a collaborative AI precision rehabilitation framework using model-based contextual Reinforcement Learning (RL) and demonstrated how it can be used to optimize longitudinal treatment plans (simplified as the timing and dose of upper extremity-based treatment at discrete time steps) [5]. In this framework, rehabilitation is viewed as a Markov Decision Process (MDP): a sequential decision-making problem in which the longitudinal rehabilitation outcomes are random variables influenced by the interventions [5]. An MDP consists of four components: (i) A set of *states* representing a patient’s motor memory and recovery status, (ii) a set of *treatments* defined as the dose (possibly for different types) of rehabilitation at a time step, (iii) an unknown transition function that gives the probability of the patient transitioning to a next state given a current state and a treatment, and (iv) a reward function quantifying the resulting functional improvement at each time step. The goal of RL in an MDP is to learn a policy, i.e., a decision rule, to select treatments in each state that will maximize the total reward, which involves, for instance, both short- and long-term functional improvements.

This RL-based system involves the four key components for Precision Rehabilitation outlined above [5]. First, the system comprises a collaborative objective module that allows the clinician and patients to set the rehabilitation goals based on desired short and long-term outcomes and any constraints (or preferences). The module ensures that the RL agent’s objective is aligned with the rehabilitation goals by transforming its reward function. Adjustments can be made for both “hard” constraints and the reward function weighting.

Second, a patient model is employed to forecast individualized, time-dependent recovery processes given a treatment plan. We used a Bayesian approach to seamlessly account for *aleatoric uncertainty* (i.e., inherent randomness in a process or data) in outcomes through interval predictions and *epistemic uncertainty* (i.e., uncertainty arising from lack of knowledge) about each patient through interval estimates of patient-specific model parameters. In addition, the model included contextual information, such as stroke type, age, and lesion location, to facilitate information-sharing between patients when learning individualized models.

Third, a treatment planner is used to determine near-optimal, patient-specific policies (or decision rules) to assign rehabilitation doses (in hours) based on the patient’s status at each time step. In our initial work, we used dynamic programming to identify a (near-)optimal policy that maximizes the short- and long-term predicted outcomes given a patient model and any user-defined constraints. Since the ground-truth (or the best) patient model is unknown, the model used for planning is selected via Thompson sampling (i.e., sampled according to the Bayesian posterior distribution) to efficiently balance exploitation and exploration.

Fourth, to allow model updating, the system uses a database of all past and current patients, leading to reduced uncertainty level in the predictions and better treatment decisions (especially for new patients with little rehabilitation data). Notably, as new data arrive at every time step, the system continuously learns better models and refines its treatment plans both at the patient and population levels. Simulations using a chronic stroke model, developed with data from two clinical trials [32, 33], suggest that this AI agent becomes increasingly effective with more participants [34] and can improve motor functions more than fixed uniform, decreasing, or increasing dose schedules [5].

## Examples of AI in rehabilitation

AI has powerfully been used in other domains, such as to improve personalized care for individuals with cancer (see "introduction") and to stratify patients for more efficient and effective therapeutic trials, as has been done recently with a therapeutic drug for Alzheimer’s disease [35]. However, collaborative AI frameworks of precision rehabilitation are still currently emerging. Thus, here, we review how AI has so far been applied to address domain-specific complex rehabilitation challenges. Rapid advances in AI have enabled applications ranging from diagnostics to data processing to tailoring interventions. In this section, we review four applications of AI in rehabilitation in: (1) biomechanical movement analysis, (2) neuroimaging, (3) robotic therapy assistance, and (4) brain computer interfaces. The developments in each of these areas may be integrated into a larger collaborative AI model for precision rehabilitation in the future.

### AI for clinically accessible biomechanical movement analysis

Traditionally, marker-based motion capture has been used for biomechanical assessments in research and select clinical settings, such as decision-making in orthopedic surgery. However, high costs and logistical constraints have made routine use impractical, leaving clinical outcome assessments for gait and balance reliant on subjective assessments, such as stopwatches and visual judgment.

Recent AI-powered methods now incorporate the use of wearable sensors to measure movement in both clinical and real-world settings, as well as synchronized multiview video and monocular video. The video-based methods are driven by recent advances in computer vision, specifically human pose estimation (HPE). While early HPE approaches for monocular video could only identify body part locations, new methods incorporate multiple views for 3D biomechanical modeling, achieving accuracy comparable to marker-based systems with shorter setup times and greater accessibility [36–40]. It is now possible to track every joint in the hand with an accuracy of several millimeters, providing a quantitative characterization of arm and hand impairments that were previously impossible [41]. Recent advances even allow fitting 3D biomechanical models to monocular video captured by a smartphone, making quantitative biomechanical assessments even more accessible [42].

However, while these techniques enable longitudinal quantification of movement impairments, it is not yet clear how to use such data to improve rehabilitation outcomes. Emerging research studies may suggest an avenue. For example, spatiotemporal gait parameters are associated with fall risk, a major contributor to mortality and morbidity. Integrating gait analysis into routine clinical visits and home assessments could identify individuals at risk of falls and enable early interventions. However, workflow integration issues, such as electronic medical record compatibility, must be addressed before this technology can be widely used. Furthermore, the technology must be designed to be easy for clinicians to use and to produce reports that they find useful. Additionally, a lack of longitudinal movement data currently limits our ability to identify patient subgroups for targeted interventions. Combining these large clinical movement datasets, which can now be collected, with long-term outcomes is a promising avenue to let data guide our next generation of clinical assessments, as discussed under the Causal Framework for Precision Rehabilitation above.

### AI for brain imaging in neurorehabilitation

A second area where AI has been integrated into diagnostic tools for neurorehabilitation is neuroimaging. In particular, brain imaging has been widely used to diagnose and identify biomarkers of neural injury and neurodegenerative disorders. Brain imaging contains hundreds of thousands of voxels (or 3D volumetric pixels) of data, and AI is well-suited for processing such large datasets. Current applications of AI in neuroimaging include: (1) segmenting brain features of interest [43–45], (2) quantifying brain measures that are difficult to assess visually [46, 47], and (3) generating synthetic high-resolution images from low-resolution clinical scans [48, 49].

Traditionally, manual segmentation of brain structures such as the hippocampus (to quantify hippocampal volume, a biomarker of neurocognitive disorders), or a stroke lesion (to quantify overlap with key structures, such as the corticospinal tract or CST), is labor-intensive and requires extensive neuroanatomical expertise. Recent research efforts have focused on automating these processes using AI algorithms trained on manually segmented images. AI-based tools for automated hippocampal [45], whole brain segmentation [50, 51], and lesion segmentation [44, 52] among others, have been developed and tested in clinical populations at a rapid pace, improving researchers’ capacity for large-scale brain analyses.

AI has also enabled the extraction of new brain features beyond human observation. One example is brain age, a neuroimaging-based measure that estimates a person’s age based on their brain features [53]. Brain age algorithms are trained on thousands of healthy brains with labeled chronological ages to predict the ages of new people. The difference between predicted and chronological age (brain predicted age difference or brain-PAD) is thought to be an indirect measure of brain atrophy [53], correlating with conditions such as Alzheimer’s disease [54], depression [55], schizophrenia [56], traumatic brain injury [57], and risk of mortality [58]. Recent studies have begun to link brain-PAD to neurorehabilitation [46]. Brain-PAD after a stroke correlates with the severity of sensorimotor impairment and the size of lesion damage at 90 days post-stroke, and moderates the impact of CST lesion damage on sensorimotor outcomes, such that better brain-PAD is linked to better sensorimotor outcomes, despite large lesion damage [46]. Finally, a recent study demonstrated the utility of brain age for disease stratification in Huntington’s disease [59], which could impact the efficiency of clinical trials.

Finally, in the last two years, generative AI—that is, AI designed to generate new information has further transformed neuroimaging. Tools like SynthSR [48] and its successors [49, 60] can convert low-resolution clinical scans into high-resolution images, allowing the repurposing of vast amounts of clinical imaging data, which had previously been considered too low-resolution for scientific use. Generative AI has enormous potential to improve imaging-based assessments used in precision rehabilitation models. In the future, low-resolution clinical MRIs could be used with an AI-assistive tool to generate detailed projections of brain health and associated prognoses for clinicians to help guide treatment decisions.

### AI for robotic therapy assistance

With the increasing prevalence of motor impairments caused by neurological diseases, coupled with limited human and financial resources, many patients receive inadequate physical therapy. Over the past decades, robotic systems like the Lokomat, designed for walking training, have been developed to support therapists and increase therapy time. These rehabilitation robots can precisely control training, presented as engaging serious games, and assess performance. In addition, serious games, either on their own or in conjunction with virtual reality, have also been integrated to enhance neurorehabilitation [61]. In recent years, robot systems have been simplified to provide assistance in simple settings and at home [62–64]. However, how can robotic systems be controlled to address the varying impairments and therapy needs of individual patients? Can AI enable the creation of robotic therapists that can dynamically adapt therapy, as human therapists do?

In cases of severe impairments, robots can guide the limb through predefined movements to stretch muscles, enhance passive range of motion, and help reduce spasticity. However, when patients become able to move by themselves, too much movement guidance induces passivity and this can alter an individual’s motor behavior and recovery [65, 66]. Therefore, adaptive techniques have been developed for patients who have regained some movement ability, regulating robotic assistance to support their efforts while still challenging them to encourage proactive learning [65, 67]. These methods have been successfully applied, for instance, in walking assistance systems like the Lokomat [68] and could be used to correct for compensatory behaviors [69].

Despite their benefits, these adaptive approaches restrict patients’ movements to predefined trajectories. Recent advancements leverage differential game theory to overcome this limitation by assisting patients in executing their own intended movements [70, 71]. This innovative approach enables robotic systems to (i) identify the patient’s sensorimotor condition in real time, and (ii) dynamically adapt interaction behavior, offering a range of support from full assistance to collaborative effort sharing or even competitive engagement. By tailoring the level of support and challenge to the patient’s condition, this method can promote optimal training, and pave the way for AI-driven therapy systems that emulate the adaptability and responsiveness of human therapists.

### AI and brain-computer interfaces for rehabilitation

Brain-computer interfaces (BCIs) illustrate both the promise and the challenges of applying AI in precision rehabilitation, as they translate brain activity into commands for external devices, such as assistive robots or exoskeletons [72, 73]. While early BCIs relied on simple linear classifiers, recent advances in computing power and data availability have enabled the integration of AI, significantly enhancing neural decoding [74]. By improving the accuracy of brain signal decoding and increasing system responsiveness, AI facilitates more effective communication between the brain and external devices, overcoming traditional hurdles such as slow information transfer and inconsistent signal clarity. This progress highlights AI’s potential to improve performance in assistive applications, but it also raises questions about how to design systems that foster genuine restorative effects rather than merely adapting the machine to the user.

*Assistive BCIs and AI.* Assistive BCIs enable users to control external devices, such as assistive robots, prosthetic limbs, communication tools or spinal cord stimulators. Through operant conditioning [75] and feedback learning, BCI users learn to modulate their brain activity to control external devices such as prosthetics, communication tools, or spinal cord stimulators [72]. Furthermore, integrating direct electrical stimulation of brain tissue in conjunction with input from force sensors can help re-establish sensory feedback. This bidirectional interaction approach enhances the functionality of prosthetic limbs [76]. AI-enabled assistive BCIs are used in robotic exoskeletons for patients with paralysis, restoring functions such as grasping or walking [77]. AI can also enhance systems by providing artificial sensory feedback through electric stimulation, dynamically adapting to environmental changes. The combination of AI within the context of bidirectional interaction in assistive BCIs promises to allow for enhanced motor control, increased adaptability, and potentially even the restoration of proprioception.

The field of AI robotics, including natural language processing and vision-guided robotics, has seen phenomenal growth in recent years. Thus, it is possible that robots that are responsive to high-level verbal commands will be an important assistive tool for people with severe motor impairments and may avoid the need for invasive BCI for people with intact speech. For individuals who are unable to speak but have intact language brain areas, BCIs for speech decoding, which have seen significant improvements in performance over the past few years, could provide an important domain of assistive technology.

*Restorative BCIs and AI.* Restorative BCIs aim to rehabilitate motor, sensory, or cognitive functions by promoting neuroplasticity through contingent feedback between brain activity and external stimulation [78]. The integration of AI into these systems, although still in its early stages, holds considerable promise for refining neural signal decoding, adapting task difficulty, and optimizing stimulation patterns in real time. Yet, the role of AI in restorative BCIs is not without challenges: most existing approaches prioritize adapting the computer to the user to maximize decoding accuracy, which may improve short-term control but reduce the brain’s incentive to adapt, thereby limiting restorative potential. A key task for collaborative AI in this domain is therefore to strike a balance between machine adaptation and patient-driven learning, ensuring that AI augments rather than replaces the neural processes underlying recovery. Concrete examples include AI-enhanced BCIs that couple motor imagery with robotic assistance or functional electrical stimulation to drive motor relearning, as well as neuroprosthetic systems that provide artificial somatosensory feedback to reinforce adaptive changes. Beyond motor rehabilitation, AI-based systems are being investigated for modulating brain activity in conditions such as epilepsy and depression [79]. In the longer term, collaborative AI system could serve as a decision-support layer, recommending individualized BCI protocols that are tailored to a patient’s impairment profile, therapeutic goals, and progress, thereby embedding BCIs into a broader precision rehabilitation framework.

## Making AI-based precision rehabilitation a reality

### The need for large, diverse and high-quality data

AI-based neurorehabilitation is in its early stages. The three frameworks described in Sect. 2 are either conceptual (CFPR), focus on select aspects (NeuroAIreh@b; cognitive rehabilitation based on questionnaires and discrete action sequences rather than complex body function rehabilitation), or are in the early stages of implementation (Contextual Model-based Reinforcement Learning), while the applications of AI to specific rehabilitation needs are rapidly emerging. To make precision rehabilitation a reality, we must integrate these AI-based frameworks and solutions with real-world data to develop accurate models of rehabilitation outcomes. A major challenge in achieving this is the need for extensive, detailed datasets that capture critical rehabilitation-specific variables necessary to train and test accurate, reliable, and generalizable algorithms. However, generating these datasets is difficult. Clinical electronic health records (EHRs) provide large, real-world datasets but often lack detailed rehabilitation-specific factors, leading to oversimplified models. Conversely, research datasets rich in relevant variables are typically small (e.g., *N* < 100), use various outcome measures across the ICF domains, and only a few are publicly accessible. Although the revised NIH Data Management and Sharing policy aims to increase the availability of shared research datasets, harmonizing data across studies remains difficult due to differences in data management practices, inclusion/exclusion criteria, choice of outcomes, study timelines, type of interventions, and other methodological inconsistencies.

Currently, researchers must contend with data from multiple heterogeneous sources that often lack comprehensive metadata and clear encoding of participant data, as well as consistent data element specifications. This data fragmentation, coupled with challenges in generating large single-site datasets, has created a data scarcity problem, hindering progress in precision medicine [3]. Centralized efforts, such as by the new NIH-funded Data Science and Analytics for Precision Rehabilitation (DAPR) Center, aim to establish comprehensive data standards and infrastructure that facilitate data integration across multiple sources (https://dapr.usc.edu/). DAPR builds upon initial harmonization efforts in the rehabilitation field from domain-specific areas, such as the ENIGMA Stroke Recovery Working Group, which has brought together high-resolution MRI and behavioral data from over 2000 individuals across more than 65 different stroke research studies worldwide [80]. This collaborative work has resulted in numerous scientific advances by providing large, diverse datasets for well-powered statistical analyses that no single researcher could collect efficiently on their own [46, 47, 81–83]. DAPR aims to extend this type of approach to a large scale, across multimodal domains of research, to enable rehabilitation researchers to easily discover, reuse, and analyze data for precision rehabilitation models. This is in line with recent papers and efforts encouraging the development of large data repositories of clinical data [84, 85].

A promising approach is to develop pipelines that harmonize both existing and newly collected data, from both research and clinical sources, through universally accepted common data models and data standards, such as NINDS CDE [86] and OMOP [87, 88]. These common data models, or conceptual representations that organize data elements and define how they relate to each other and how they are stored, as well as data standards—agreed-upon guidelines that dictate how data should be structured, recorded, and exchanged—can enhance the consistency, interoperability, and data quality across sites and users. The adoption of a standardized common data model across rehabilitation will enable significant progress in precision rehabilitation.

Standardized data will also facilitate federated learning, a technique that utilizes multiple decentralized datasets for model training while keeping data localized [89, 90]. This supports data privacy and security, which is crucial for clinical and EHR data containing sensitive patient information. Multiple research groups are exploring federated learning for gait analysis across different clinical populations [91, 92], highlighting the growing interest in federated learning for rehabilitation AI. However, developing the necessary infrastructure and algorithms for distributed model training remains a substantial challenge.

Although large-scale efforts to harmonize data across rehabilitation are currently underway, there are short-term targets that readers can pursue to make this long-term vision a reality. At a very basic level, aligning one’s dataset with a current common data model, such as OMOP or NINDS CDE, will help to prepare the data for future integrations. Such work will also facilitate collaborations that individuals can initiate with one another (e.g., collaborating between a few individual research labs to pool together a joint dataset). In addition, researchers could utilize existing electronic health records datasets from their local institutions, which are already rich sources of clinical, real-world data that can provide initial forays into developing and testing models. Those without access to EHR datasets could access other open-source datasets; curated rehabilitation-specific datasets can be searched on ReproRehabDB (https://reprorehabdb.usc.edu/), on NIH-funded repositories such as the NICDH Data and Specimen Hub (DASH; https://dash.nichd.nih.gov/), or on modality-specific repositories such as OpenNeuro (https://openneuro.org/) for neuroimaging data, or on the Mobilize Center (https://mobilize.stanford.edu/data/available-datasets/) for kinematic data.

Finally, simulated datasets are becoming increasingly used to develop precision medicine algorithms [93], as they can help overcome data scarcity and address privacy and bias issues. Synthetic data are generated by machine learning models that learn from real research or clinical data to create new, privacy-preserving datasets that mimic the statistical properties of the original data. Synthetic data can then augment real patient data by generating large, diverse virtual patient cohorts to train AI models for targeted treatments. For instance, a reinforcement learning agent can learn an optimal “policy” by continuously interacting with the virtual patient environment. The policy can then be fine-tuned with smaller, real-world datasets.

### Expanding data sources to improve the effectiveness of AI for neurorehabilitation

In addition to standardizing existing data, new data sources should be incorporated. AI-powered video analysis can provide detailed movement quantification during rehabilitation and in-home settings. Wearable sensors and smartphone- or smartwatch-based motion tracking provide further insights into real-world functional performance, eliminating the need for constant video observation. However, managing these data streams for thousands of patients requires substantial infrastructure investment, and further research is needed to determine the best way to leverage them for therapeutic decision-making.

Recent advances in technology also enable the real-time monitoring of behavior as people move around, interact with objects, and perform exercises and activities in their natural environment. Sensorized objects, such as the FitMi [94] and GripAble [95] systems, can be used for home exercise programs through serious games, providing their users feedback on the applied force, action duration, rate of repetitive movements, and accuracy, while also offering researchers robust kinematic and kinetic data on trial-to-trial performance.

Ecological momentary assessment (EMA) and ecological momentary intervention (EMI), originally developed in behavioral medicine to track fluctuations in pain, can be effectively adapted for rehabilitation research. These methodologies involve delivering 2 to 6 smartphone-based prompts throughout the day, prompting patients to complete brief surveys assessing pain, paretic limb function, and contextual factors such as mood, activity, and social interactions. Recent research also highlights self-efficacy as a key mediator in the relationship between balance, walking performance, activity, and participation post-stroke [96]. Importantly, EMA can be combined with accelerometry to assess paretic limb use at home in stroke survivors [97, 98], where social context plays a crucial role [99]. The limited transfer of rehabilitation-acquired skills to real-world settings–the so-called “Capacity to Performance” disconnect [100]— is a complex, multifactorial challenge that can be addressed through integrated qualitative and quantitative approaches, enabling more personalized interventions [101–103]. This iterative process—leveraging body-worn sensors, real-time EMA/EMI data, and a self-learning collaborative AI system—holds significant promise for the future of precision rehabilitation.

Finally, the models will only be as good as the quality of the data collected. As remote monitoring capabilities, EHR integration, and learning health systems advance, standardization procedures and related clinician training will be essential [104]. Ensuring high-quality data collection and management is a critical step toward realizing the full potential of AI-based precision rehabilitation.

### Ethical dimensions of AI in rehabilitation

#### The importance of trustworthy AI

For collaborative AI to be effectively adopted in rehabilitation, it must be trustworthy. Without trust from patients, families, clinicians, and other stakeholders, adoption will remain limited. In AI, trustworthiness encompasses several key factors, including explainability (‘Why did the model make a decision?’), confidence (‘How certain is the model about its decision?’), and fairness (‘Are there any biases in the model’s decision?’). We now briefly elaborate on these factors.

*Explainability* in AI, also known as XAI, is a field of rapidly growing methods for helping humans explain AI models [105, 106]. While some statistical models, like linear regression, are inherently interpretable, many AI models used in clinical applications, such as neural networks, operate as “black boxes” – where the input generates a prediction, but the reasoning behind the prediction is unclear. Whereas neural networks often have high accuracy, the challenge lies in making these models explainable by identifying which input features were most important in generating a specific output. In contrast to these “black-box” models, which require large datasets for development, theory-driven “grey-box models” (i.e., those with a structure motivated by theory and parameters estimated from patient data) can be based on smaller datasets and offer good explainability. Analysis of the parameters in such interpretable models can help the clinician make informed decisions about therapy. For instance, in our previous model of the change in an upper extremity functional outcome as a function of motor therapy [34], if the estimated decay parameter is high, more frequent “booster” sessions may be needed to maintain function. We note, however, that such grey-box models often have relatively worse predictive performance than black-box models due to simplifying assumptions, such as time invariance and linearity. In any case, to make meaningful progress, explainability should encompass key dimensions such as human understandability, consistency, completeness, and model agnosticism, ensuring that the model’s decisions are transparent, trustworthy, and actionable in real-world applications.

*Confidence.* The quantification of uncertainty in AI model predictions is arguably a more tractable problem than the issue of explainability [107]. Uncertainty can be quantified during model training or at decision time, where a point prediction is accompanied by confidence intervals (e.g., 95%) or presented as a probability (e.g., a 90% chance of a positive outcome) [107, 108]. These methods can be readily implemented, for instance, via Bayesian modeling (see the third framework above and [34]). A major challenge is how clinicians or patients interpret uncertainty. In other words, if a given treatment is predicted to have a 50–60% chance of a positive outcome, should it be implemented, especially if it is costly or invasive? Simulations with different treatment scenarios of patient models that generate both point predictions and confidence intervals (Fig. 1, component 2), under different reward functions (Fig. 1, component 1) can help decide between treatments.

*Fairness. *Finally, maintaining the fairness of AI models is a well-known challenge for their practical use, as biases, such as those related to ethnicity, have been identified in existing systems [109, 110]. Biases arise from the systematic under- or over-representation of certain aspects of the training data or input features, leading to inaccurate decisions or poor generalizability. In particular, people with disabilities are often underrepresented in many datasets, raising concerns about bias in AI models used in rehabilitation [111, 112]. The most effective way to reduce demographic biases is to ensure that training datasets are representative of the general population (or the specific clinical population in question). When truly representative training data are unavailable, AI techniques such as transfer learning (where a pre-trained model from a large dataset is fine-tuned on a smaller, more targeted dataset) or simulated datasets (see above) offer promising alternatives by leveraging existing datasets, even if they are initially unrepresentative.

While challenges remain for the practical implementation of trustworthy AI, approaches to explainability, confidence, and fairness can have an immediate application in data quality assurance and control to ensure the maintenance of high-quality datasets for research efforts in optimizing rehabilitation programs. For example, even if we cannot robustly validate whether an XAI method has identified the ground truth features for predicting treatment response, these methods can readily identify artifacts and outliers in data that could negatively impact model performance and should be removed.

#### Responsibility and privacy

Beyond measures of trustworthiness, additional ethical considerations must also be addressed. One major issue is responsibility: if an AI-based treatment leads to poor outcomes, who is accountable—the clinician, the software developer, or the AI researchers? In the case of collaborative AI, does the responsibility ultimately lie with the clinician? If so, will the clinician need to constantly manually check the AI’s data and interpretations, and will this become more time-intensive than not using the AI-based data insights? Another ethical challenge involves data privacy, as AI models typically require vast amounts of sensitive biomedical data, necessitating robust information governance. However, as AI is a priority across multiple domains, not just rehabilitation, the collective efforts of researchers in healthcare and biomedicine will help develop solutions. Rehabilitation scientists should be prepared to contribute to this broader effort.

### Promoting adoption of AI-based precision rehabilitation in the clinic

AI-based precision rehabilitation is designed to improve outcomes and efficiency. However, a proof of efficacy is insufficient for clinical adoption. In fact, even highly performing AI systems often face resistance from clinicians. Research on AI adoption in healthcare has identified three primary barriers [113]: (1) Little direct benefit to the clinician using the tool – for instance, as the patient model will need to incorporate baseline data and initial outcome measures recorded during the inpatient phase, inpatient clinicians who may enter these data may not see the long-term benefits of AI systems for their patients (e.g., in the outpatient or chronic phase of case); (2) Additional workload to the clinician– for instance, to enter structured data in the system; and (3) Loss of autonomy, as clinicians may fear that AI-driven recommendations could limit their decision-making authority.

To mitigate these issues and increase clinical adoption, we propose three possible solutions: (1) First, AI should focus on reducing the clinician’s workload by automating documentation systems and routine tasks, allowing clinicians to focus on patient care. This was termed the Gift of Time by Eric Topol [114], who suggested that AI should relieve clinicians from repetitive administrative tasks, allowing them to spend more meaningful time with patients. (2) Second, the reporting burdens should be minimized; AI should enable automatic data collection from sensors and seamless integration with electronic health records instead of requiring humans to perform manual data input and scrubbing. We posit that the adoption of AI will require AI tools that are easier to use than existing tools, which are often as simple as clicking a stopwatch. (3) Third, preserving clinician autonomy via collaborative AI is critical, as it will offer suitable plans without overriding clinician judgment, much like navigation apps suggest multiple routes but leave the decision to the driver. The clinicians will be able to simulate predictive models for different preferred plans, allowing them to make their own decisions and providing a form of interpretability that supports trustworthiness. Regardless of how an AI-driven plan is determined (whether by the AI system, collaboratively with clinicians, or entirely by human judgment informed by AI-derived data), continuous data collection will enhance future predictions. In addition, the system may learn some novel near-optimal treatment plans from expert clinicians, further refining rehabilitation approaches.

## Discussion and future directions

Rehabilitation is a uniquely complex domain for AI, requiring the integration of numerous biological, social, and cultural factors, as well as diverse goals, outcomes, and models. It is likely that the development of collaborative AI for rehabilitation will be iterative, where advancements in specific areas could then be extended across the field. Communication and collaboration across groups incorporating AI into rehabilitation is thus critical; open science, transparency, and the sharing of methodologies and data will drive this field forward. Additionally, no single organization or discipline can alone tackle the complex challenges analyzed above. Collaborations across rehabilitation scientists, clinicians, engineers, computer scientists, and implementation scientists are vital to developing solutions that can be effectively deployed and used in the real world.

In our roadmap for a collaborative AI rehabilitation system, we proposed an “online” clinical decision system that learns to generate optimal treatments by interacting with patients. The system tries different treatments, the patient generates an outcome, and the system adjusts its strategy accordingly, making better treatments more likely to be selected. In contrast to such an online system, offline systems [115, 116] are often preferred in treatments where incorrect actions can result in significant harm or even death, such as whether or not to initiate mechanical ventilation or the treatment of sepsis [117]. Offline systems learn from a fixed, pre-collected dataset of past experiences, without any interaction with the patient during training. However, a significant weakness of offline RL systems is that they cannot discover or adjust treatment plans for the current and future patients. We suggest that in rehabilitation, an online approach, as we proposed, is acceptable because each session poses little risk, especially if we ensure that treatment constraints, such as weekly dose, can be modified at any time, and that the clinician retains autonomy for adjusting the plan and making the final treatment decision. Other online systems have been developed in the field of digital health, which is also characterized by low risk to users. For instance, an online algorithm was devised to optimize the delivery of prompts to encourage participants to engage in oral self-care behaviors [118]. Future research should compare the effectiveness of online versus. offline systems and the ease of implementation in rehabilitation practice.

In addition, rapid advances in large language models (LLMs) for medicine present growing opportunities. For example, in a double-blinded study, an LLM trained for medical conversations called Articulate Medical Intelligence Explorer (AMIE) outperformed human clinicians in making a differential diagnosis through a chat interface [119], and helped with chronic disease management by referencing clinical practice guidelines, on which it was found to be non-inferior to primary care providers in a blinded study [120]. AMIE chat interface has been further extended to support multimodal processing, such as dermatology photos or EKGs, where it also performed very well [121]. However, this work does not yet apply to rehabilitation, although it may in the future. A recently developed rehabilitation-specific model is BiomechGPT, a multimodal LLM fluent in biomechanics. Its performance was tested on several clinically meaningful tasks, including identifying gait impairments and their possible etiologies, as well as classifying activities such as the timed-up-and-go and walking [122]. This offers a promising avenue for extending LLMs to perform a wide range of rehabilitation tasks, such as scoring clinical outcome assessments. Our proposed collaborative AI system (Fig. 1) could benefit from integration with such LLM systems. For instance, the component “Collaborative goal-setting” could be augmented with an LLM by integrating data from the current patient and clinical guidelines; the system would then, in collaboration with the clinician, determine the goals and high level treatment plan (such as “the patient would benefit from neuromodulation over the contralesional motor cortex in conjunction with an upper extremity rehabilitation to improve arm function”), which would be sent to the AI-treatment planner (component 3) to determine the details of the sequential treatment plan.

However, as models progress, it is also crucial to identify AI’s limitations, as its capabilities are not yet fully understood, particularly in rehabilitation, where it may be useful or consistently fail. AI is not meant to replace humans or decrease their performance, but rather assist them and extend their abilities. Reports of AI systems unintentionally increasing workload or reducing human interaction, such as restaurant employees monitoring AI-driven automation instead of engaging with customers, highlight potential pitfalls. To avoid similar setbacks in rehabilitation, the efficiencies gained from AI integration must not be counterbalanced by an increase in low-value administrative tasks. Instead, AI should both improve outcomes and simplify processes, allowing therapists to dedicate more time to patient care and meaningful human interaction.

Finally, once precision rehabilitation AI models have been validated with real-world data, significant effort will be required to translate them into clinical practice. Ensuring that the models are trustworthy and acceptable to the people using them will be crucial (e.g., clinicians, patients, caregivers, and even insurers). Implementation science and pre-implementation studies should actively assess perspectives, concerns, and potential barriers to adoption throughout the model development process. Learning healthcare systems, which are health systems that utilize data to enhance patient care, offer an ideal environment for testing AI-driven rehabilitation approaches by providing large, real-world datasets for model training and iterative refinement. These systems enable the development of precision rehabilitation systems that can truly enhance the clinician’s capability to improve their clients’ outcomes. Ultimately, collaborative AI promises to transform rehabilitation by leveraging vast, diverse datasets to create individualized digital profiles. These profiles can first be used to simulate the effects of rehabilitation interventions in silico, maximizing impacts before real-world implementation. By guiding personalized rehabilitation strategies based on empirical evidence, collaborative AI holds promise as a powerful tool to help clinicians advance patient care and improve long-term outcomes.

## Data Availability

No datasets were generated or analysed during the current study.
